# A descriptive study of biological and psychosocial factors associated with body mass index for age, in adolescents attending an outpatient department at Weskoppies Psychiatric Hospital

**DOI:** 10.4102/sajpsychiatry.v23i0.973

**Published:** 2017-08-31

**Authors:** Kgomotso R. Ncube, Nadira Khamker, Deborah van der Westhuizen, Thea Corbett

**Affiliations:** 1 Department of Psychiatry, Faculty of Health Sciences, School of Medicine, University of Pretoria; 2 Department of Statistics, University of Pretoria, South Africa

## Abstract

**Objective:**

To describe biological and psychosocial factors associated with body mass index (BMI) for age in adolescents attending an outpatient department at Weskoppies Psychiatric Hospital.

**Methods:**

A total of 50 adolescents participated in a convenience sampling research study. BMIs were calculated using their weights and heights to distinguish different weight categories based on the 2007 World Health Organization (WHO) growth charts. Based on their BMIs, participants were categorised as underweight, normal body weight, overweight and obese. The association between the BMIs of the biological parents and their adolescent children was investigated using the Fisher’s exact test. The data collection included adolescents’ demographic information, psychiatric diagnosis, psychiatric medication, nutritional intake, eating habits and the intensity of physical activity such as sports, leisure and sedentary behaviour.

**Setting:**

The study was conducted at Weskoppies Psychiatric Hospital’s adolescents outpatient department.

**Results:**

Of the participants, 72% were males. Forty-eight per cent of all the adolescents had a normal BMI, mostly of black African descent. When comparing the adolescents’ BMI with that of their biological mothers, 50% of those who were obese also had mothers who were mostly obese (53.8%). The Fisher’s exact test indicated a statistically significant association between the BMI categories of mothers and those of their adolescent children (Fisher’s exact test, *p* = 0.032). Despite the above association, no significant association could be found regarding their nutritional intake and eating habits. Also, no significant association was found between the adolescents’ BMIs and the use of psychotropic medication, as compared with other previous studies. Furthermore, no association could be found between adolescents’ BMI categories and the level of intensity of physical activity such as sports and leisure activities or sedentary behaviours.

**Conclusion:**

This study supports previous findings that a significant association exists between maternal and childhood obesity. The association between BMI and psychotropic medication, nutritional intake and eating habits, and level of physical activity could not be confirmed in our study. The study results were limited by the small sample size and the convenience sampling method. Although this was only a descriptive study, it highlighted the complexity of biological and psychosocial factors involved in weight gain. Further studies are needed to explore the interplay of physical and environmental risk factors for childhood obesity, as well as to ensure early identification and education of patients and their families to prevent development of obesity.

## Introduction

Obesity has long been a major health concern among adults, but its increasing prevalence rates and associated health risks in children and adolescents over the last three decades have made it a public health priority.^1^ Childhood obesity has reached epidemic proportions worldwide despite major efforts to promote weight reduction, with more than 75% of overweight and obese children living in low and middle income countries, particularly in urban settings.^2^

The worldwide prevalence of childhood obesity (from birth to 5 years) had progressively increased from 4.2% in 1990 to 6.7% in 2010.^3^ The World Health Organization (WHO) has since 1998 considered obesity to be a global epidemic which not only affects developed countries.^3^ In 2010, 43 million children under the age of 5 years, of which 35 million were in developing countries, were overweight and obese, and 92 million were considered to be at risk of becoming overweight.^3^ In the United States (US) alone, between 1999 and 2000, 30.0% of teenagers aged 12–19 were identified as overweight or obese, and in 2003–2004, the prevalence increased significantly to 34.4%. Similar increases were seen in children of ages between 6 and 11 years. The 2013 Heart Disease and Stroke Statistics Update revealed that 23.9 million children (31.8%) aged 2–19 years were overweight or obese and 12.7 million (16.9%) were obese.^4^

The increasing prevalence of overweight and obesity in children and adolescents is very disturbing as childhood obesity is predictive of adult obesity.^5^ Children who are overweight and obese (body mass index [BMI] ³ 95th Percentile) are also at risk for obesity-related illnesses, which may emerge in childhood. Of particular concern are the increasing numbers of youth exhibiting Type 2 diabetes mellitus, dyslipidemia and hypertension which contribute to cardiovascular disease risk.^6^ Overweight and obesity in children and adolescents have been associated with increased risk of both premature mortality and adult morbidity.^7^

The Centres for Disease Control (CDC) classifies a child as at risk for overweight, if the BMI is at or above the 85th percentile and below the 95th percentile for age, meaning a child whose BMI is greater than or equal to the 95th percentile for age is considered overweight. The WHO uses similar percentiles but different labels regarding a child with a BMI between the 85th and the 95th percentile as overweight and the one with a BMI of greater than the 95th percentile as obese.^8^

Many factors have been identified to influence the development of obesity in children and adolescents, among which are increased intake of high fat foods, sweetened soft drinks, fruit and vegetable intake, sports habits and families’ medical history.^9^ The KwaZulu-Natal Department of Health guidelines have recommended regular consumption of meals with different kinds of food, consumption of fatty foods sparingly, regular consumption of protein foods and consumption of snacks with high fibre and low fat content.^10^ The Birth to Twenty (Bt20) cohort study conducted at the University of the Witwatersrand in South Africa reported an association between increased TV viewing, reduced fruit and vegetable consumption and more snacking, which leads to childhood obesity. Cross-sectional analyses have shown that fast-food intake, breakfast skipping and not eating meals together with the family have a positive relationship with obesity.^11^

A US study by Bradford has shown that the presence of parental obesity doubles the risk of a child becoming overweight as an adult. Parental obesity, especially in the mother, has been found to be the strongest of all the risk factors for the development of obesity.^12^ A South African study on nutritional status of children and obese mothers has also found that obese mothers had significantly more overweight and obese children than the non-obese mothers.^13^ Recent reviews have indicated the importance of parental involvement and parental monitoring of child health behaviours in preventing and treating childhood obesity.^1^ Family meal setting has the potential to positively impact the dietary intake of children. Research has also indicated a positive association between family meal frequency and improved dietary quality among children and adolescents. Eating out is often associated with higher fat intake and less fruit, vegetables and milk consumption.^14^

The prescription of second generation antipsychotics (SGA) has increased substantially among young patients in recent years. An Australian study has reported that 80% of increased weight gain has been observed as a side effect of SGA in young patients. This may, as a result, lead to a risk of developing Type 2 diabetes mellitus, cardiovascular morbidity and metabolic syndrome as sequelae of significant weight gain attained as a side effect of this medication.^15^

Sedentary behaviour and sleep may increase the likelihood of a child becoming overweight. Reduction of sedentary behaviour such as media screen time has been extensively researched and found to be an intervention target, as children today spend more time than ever before engaged in inactive pursuits.^16^

The development of effective programmes for the prevention of overweight and obesity in children is thus necessary to turn the tide of the obesity epidemic. Studies on the prevention of childhood obesity have shown beneficial effects of these programmes. The emphasis was on education, nutrition and physical activity, parental support and home-based interventions including improved diet and exercise.^4^

If current trends continue in adulthood, an even greater increase in obesity-related health problems and in obesity-related economic costs such as loss of productivity, disability, morbidity and premature deaths can be anticipated. The probability of transitioning successfully from obesity to normal body weight in adulthood through voluntary weight loss is low, thus prevention of obesity in children and adolescents has become a public health strategy to reduce overall prevalence of obesity and its health and social consequences.^17^

In view of these risk factors, early identification will enable educating patients and their families on ways to prevent this problem and also allow early detection and referral for further management in the vulnerable population.

The purpose of this study was to evaluate the biological and psychosocial factors associated with overweight and obesity among adolescents attending an outpatient department at Weskoppies Psychiatric Hospital.

## Methods

### Participants

The study was conducted on 50 adolescents aged 12–18 years. They were requested to participate in the study as they presented to the outpatient department for their medication follow-up. The inclusion criteria entailed patients with pre-existing psychiatric diagnosis and on psychotropic medication. Patients with diagnoses of intellectual disability and existing medical conditions other than obesity or overweight were excluded. Participants also had to be accompanied by their parents or guardians for completion of the questionnaires and consent forms. BMIs were calculated for parents and children.

### Setting

The study was conducted at the adolescent outpatient department of Weskoppies Psychiatric Hospital, a specialised psychiatric hospital.

### Study design

Convenience sampling was conducted in this descriptive study.

### Measurements

Data on the biological and psychosocial factors were collected using a diet questionnaire on nutritional intake and eating habits, and physical activity questionnaire measuring the type of activity and amount of time spent on sports and leisure activities, as well as sedentary behaviour.

Body mass indexes of the adolescent participants and their parents were calculated using their weights and heights to distinguish different weight categories based on the 2007 WHO Growth Reference chart.

Four weight categories of adolescent participants were identified: underweight, normal body weight, overweight and obese.

### Data analysis

Analysis of the data consisted of frequencies, cross tables and descriptive statistics such as means and standard deviations. The Fisher’s exact test was used to investigate the association between the BMI of the biological parents and adolescents. The statistical analysis was performed using IBM SPSS Statistics version 22.

## Ethical considerations

The study was approved by the Faculty of Health Sciences Research Ethics Committee of the University of Pretoria. Questionnaires on demographics, psychiatric medication and psychiatric diagnosis were completed by the parents or guardians accompanying the participants.

## Results

### Characteristics of the total group

A total of 50 participants were enrolled in our study. The mean age was 15 years. Of the participants, 72.0% were males and 28.0% were females. In terms of race, black African descents were in the majority (56.0%), followed by the white African descents, (40.0%). Most of the black African descendants were of normal weight (57.1%), whereas the white African descendants were found to be overweight (35.0%) and obese (35.0%).

Of the total group of adolescent participants, 4.0% were found to be underweight, 48.0% had normal body weight, 26.0% were overweight and 22.0% were obese. Of the participants, 47.2% of males and 50.0% of females had normal BMIs (see Table 1).

**TABLE 1:** Body mass index categories for both genders.

BMI category adolescents	DV_5_: Gender		Total
	Male	Female	
**Underweight**			
Number	2	0	2
Column %	5.6	0.0	4.0
**Normal weight**			
Number	17	7	24
Column %	47.2	50.0	48.0
**Overweight**			
Number	11	2	13
Column %	30.6	14.3	26.0
**Obese**			
Number	6	5	11
Column %	16.7	35.7	22.0

**Total**			

Number	36	14	50
Column %	100.0	100.0	100.0

DV5, 5th percentile speed reduction; BMI, body mass index.

Of the participants, 38.0% lived with both their parents at the time of the enrolment and were accompanied by one of the following: either their parents, mothers, fathers, or guardians, on different days of their outpatient appointments.

The BMIs of the 26 mothers who accompanied their children were as follows: 11.5% were normal, 34.6% were overweight and 53.8% were obese. The BMIs of the adolescent children of the overweight mothers were mostly normal (66.7%), whereas the BMIs of the adolescent children of the obese mothers were obese (50.0%). For the cross-tabulation of BMI categories of mothers and the BMI categories of their adolescent children, the Fisher’s exact test indicated a statistically significant association between the BMI categories of mothers and those of their adolescent children (Fisher’s exact test, *p* = 0.032). See Tables 2 and 3. The BMIs of the six fathers in the study were categorised as 50.0% normal and 50.0% overweight. The BMIs of 83.3% of the adolescents who were accompanied by their fathers were normal.

**TABLE 2:** Body mass index (BMI) category of mother compared with BMI category of adolescent cross-tabulation.

BMI category of mother	BMI category adolescents				Total
	Underweight	Normal weight	Overweight	Obese	
**Normal weight**					
Count	1	0	2	0	3
Expected count	0.2	1.0	0.8	0.9	3.0
Row %	33.3	0.0	66.7	0.0	100.0
Column %	50.0	0.0	28.6	0.0	11.5
Standardised residual	1.6	−1.0	1.3	−1.0	
**Overweight**					
Count	0	6	2	1	9
Expected count	0.7	3.1	2.4	2.8	9.0
Row %	0.0	66.7	22.2	11.1	100.0
Column %	0.0	66.7	28.6	12.5	34.6
Standardised residual	−0.8	1.6	−0.3	−1.1	
**Obese**					
Count	1	3	3	7	14
Expected count	1.1	4.8	3.8	4.3	14.0
Row %	7.1	21.4	21.4	50.0	100.0
Column %	50.0	33.3	42.9	87.5	53.8
Standardised residual	−0.1	−0.8	−0.4	1.3	

**Total**					

Count	2	9	7	8	26
Expected count	2.0	9.0	7.0	8.0	26.0
Row %	7.7	34.6	26.9	30.8	100.0
Column %	100.0	100.0	100.0	100.0	100.0

BMI, body mass index.

**TABLE 3:** Results of chi-square tests.

Variable	Value	df	Asymptotic significance (2-sided)	Exact significance (2-sided)	Exact significance (1-sided)	Point probability
Pearson chi-square	13.402	6	0.037	0.028	-	-
Likelihood ratio	14.022	6	0.029	0.042	-	-
Fisher’s exact test	11.028	-	-	0.032	-	-
Linear-by-linear association	3.150	1	0.076	0.082	0.052	0.025
*N* of valid cases	26	-	-	-	-	-

### Psychiatric diagnosis

Referring to the psychiatric diagnosis, the most diagnosed psychiatric disorders were major depressive disorder (MDD), 30%, and attention deficit hyperactivity disorder (ADHD), 36.0%. Most of the participants who were diagnosed with MDD had a normal BMI (60%). Of the participants diagnosed with ADHD, 50.0% had a normal BMI.

### Psychotropic medication

Referring to the medication history, 66.0% of participants were taking SGA on their own or in combination with one or more other psychotropic medications, compared with the 14.0% who were on first generation antipsychotics on their own or in combination with another psychotropic medications. However, there was no significant association between the various combinations of psychotropic medications and BMI categories of the participants (Fisher’s exact test value = 53.512; *p* = 0.505). Only four participants were on a SGA medication alone. Two of the four of the above-mentioned participants had normal body weight, one was overweight and one was obese.

### Nutritional intake and eating habits

Of the 88.0% adolescents who ate at normal mealtimes with the family, 45.5% had normal BMI. Although snacking before meals was reported by 90.0%, 48.9% of the adolescents still had a normal BMI. Common snack food included potato chips (80.0%), chocolates (70.0%), cookies (62.0%), ice cream (50.0%), jelly sweets (44.0%), and nuts and dried fruits (26.0%).

Of the participants, 40.0% reported not eating takeaway meals, while 28.0% did so once a week. Over half of the participants did not eat from restaurants (52.0%) and always took packed food to school (54.0%). Normal BMIs were found on 46.2% of the participants who did not eat from the restaurant, as well as on 44.4% of the participants who always took packed food to school.

Half of the participants ate in front of the TV (50.0%), while 38.0% ate at the table. Fruits and vegetables were eaten by only half of the participants (56.0% and 50.0%, respectively) and most participants in these groups had normal BMI (60.7% for fruit and 52.0% for vegetables).

Of the participants, 82.0% were supervised during meals. Over half of the parents and guardians (60.0%) reported not being worried by their children’s eating habits.

### Physical activity and sedentary behaviour

Of the sports activities, football and swimming were found to be the most participated in (19.7% each respectively). Normal BMIs were found in 24.2% of those who participated in football, and in 21.2% of the swimming participants. Household chores and bike riding were the two leading leisure time activities participated in (31.5% and 20.5%, respectively). Normal BMIs were found in 29.7% of those who performed house chores, and in 21.6% who were riding bike. With sedentary behaviour, 21.2% of participants spent time listening to music, 20.6% doing their homework and 7.1% participants spent time talking on the phone. Normal BMIs were found in 19.8% of those who spent time listening to music, 18.6% of those who did homeworks, and 7.0% of those who spent time on the phone.

## Discussion

Our study describes biological and psychosocial factors associated with BMI for age in a group of adolescents attending an outpatient department at Weskoppies Psychiatric Hospital. Most of the participants were males, and had normal BMIs. Our results also revealed that most black race descents had normal BMIs, whereas most white race descents were obese or overweight.

Most of the mothers who participated in the study were found to be obese and a large number of adolescent–children of this group of mothers were also obese. A similar association was previously reported by Bradford in a US study.^12^ Association between mother and child obesity was found statistically significant in our study according to the Fisher’s exact test.

The most common diagnosed psychiatric conditions were MDD and ADHD, and most participants with these diagnoses were found to have normal BMIs. A previous study has found meta-analytic evidence of a significant association between obesity or overweight and ADHD; however, mediational effects, causal mechanisms underlying the association, as well as the long-term effects of ADHD medications on weight status in individuals with obesity and ADHD were still to be investigated.^18^ Most participants were taking SGA alone or in combination with other medications. An increase in weight as a side effect of SGA is a known and well-studied factor which has been reported in previous studies in adults. There is a lack of studies on medications causing weight gain in children.^19^ An Australian study reported an up to 80% increase of weight gain as a side effect of SGA in young patients.^15^ Our study did not confirm an association between BMIs and psychotropic medication. It would be important in future to duplicate this finding on a large sample size, as our small sample size could not demonstrate the relationship.

A large proportion of participants ate three meals a day, some of which contained high fat and dairy contents and they also made poor snack choices and ate less fruit and vegetables, but they still had normal BMIs. This was not an expected finding, because overconsumption of high-fat food is known to lead to excessive weight gain and obesity, while consumption of fruit and vegetables is thought to be protective against obesity because of their high water and fibre contents.^19^ Most of our participants ate meals with their families and ate their breakfast. A cross-sectional analysis has also shown that breakfast skipping and not eating meals with the family have a positive relationship with obesity.^11^

Most of the participants who did not eat takeaway foods, who did not eat from restaurants and who took packed food to school, were found to have normal BMIs. Furthermore, most parents or guardians reported were not concerned about their children’s eating habits and that there was supervision during their meals.

Most adolescents who participated in sports and leisure activities had normal BMIs. Most participants with sedentary behaviour also had normal BMIs. This was not an expected finding, as sedentary behaviours are known factors with the likelihood of increasing the children and adolescents’ BMIs, because of the disruption in energy balance.

This study had several limitations. The sample size was small and, as a result, most of the factors that have been studied and known to cause obesity, could not be confirmed by our study. A large sample size is needed to explore this concept further in future. The convenience sampling study method has the potential to skew the results, as it may not be representative of the population being studied. Although we did not find some of the expected associations in our study, other factors in this study are positive and encouraging; families in South Africa are still having normal mealtimes with the family, and many do not eat from the restaurants or eat takeaways, which are all protective factors against obesity. However, different BMI groups of the participants were not calculated to compare them with the different levels of overall activities, sedentary behaviours and eating habits.

Obesity has become a problem in the 21st century. Patients and their families need to be educated on the means of prevention and management of obesity, as this has become an urgent and complex concept.^20,21^

## Conclusion

A positive association between childhood and maternal obesity was found. This has been reported in previous studies. The association between BMI and psychotropic medication, nutritional intake, eating habits and physical lifestyle could not be confirmed in our study. Being a descriptive study only, the study highlighted the importance of biological and psychosocial factors involved in weight gain and the necessity for more detailed and planned studies to address the risk factors of obesity.
